# Decreased Levels of Circulating Carboxylated Osteocalcin in Children with Low Energy Fractures: A Pilot Study

**DOI:** 10.3390/nu10060734

**Published:** 2018-06-06

**Authors:** Janusz Popko, Michał Karpiński, Sylwia Chojnowska, Katarzyna Maresz, Robert Milewski, Vladimir Badmaev, Leon J. Schurgers

**Affiliations:** 1 Department of Pediatric Orthopedics and Traumatology, Medical University of Białystok, 15-089 Białystok, Poland; jpopko@umb.edu.pl (J.P.); gufkarp@gmail.com (M.K.); 2 Faculty of Health Sciences, Lomza State University of Applied Sciences, 18-400 Łomża, Poland; schojnowska@pwsip.edu.pl; 3 International Science &Health Foundation, 30-148 Krakow, Poland; katarzyna.maresz@nutricon.eu; 4 Department of Statistics and Medical Informatics, Medical University of Białystok, 15-089 Białystok, Poland; robert.milewski@umb.edu.pl; 5 American Medical Holdings Inc., New York, NY 10314, USA; vebadmaev@attglobal.net; 6 Department of Biochemistry, Cardiovascular Research Institute Maastricht, University Maastricht, 6200 MD Maastricht, The Netherlands

**Keywords:** children, bone low energy fractures, vitamin K deficiency

## Abstract

Objective: In the past decades, an increased interest in the roles of vitamin D and K has become evident, in particular in relation to bone health and prevention of bone fractures. The aim of the current study was to evaluate vitamin D and K status in children with low-energy fractures and in children without fractures. Methods: The study group of 20 children (14 boys, 6 girls) aged 5 to 15 years old, with radiologically confirmed low-energy fractures was compared with the control group of 19 healthy children (9 boys, 10 girls), aged 7 to 17 years old, without fractures. Total vitamin D (25(OH)D3 plus 25(OH)D2), calcium, BALP (bone alkaline phosphatase), NTx (N-terminal telopeptide), and uncarboxylated (ucOC) and carboxylated osteocalcin (cOC) serum concentrations were evaluated. Ratio of serum uncarboxylated osteocalcin to serum carboxylated osteocalcin ucOC:cOC (UCR) was used as an indicator of bone vitamin K status. Logistic regression models were created to establish UCR influence for odds ratio of low-energy fractures in both groups. Results: There were no statistically significant differences in the serum calcium, NTx, BALP, or total vitamin D levels between the two groups. There was, however, a statistically significant difference in the UCR ratio. The median UCR in the fracture group was 0.471 compared with the control group value of 0.245 (*p* < 0.0001). In the logistic regression analysis, odds ratio of low-energy fractures for UCR was calculated, with an increased risk of fractures by some 78.3 times. Conclusions: In this pilot study, better vitamin K status expressed as the ratio of ucOC:cOC-UCR—is positively and statistically significantly correlated with lower rate of low-energy fracture incidence.

## 1. Introduction

Bone fractures are common injuries during childhood and this number is increasing [1,2,3,4,5,6]. It has been estimated that half of all boys and one third of all girls sustain a low-energy fracture (fall from the patient’s own height or a fall during team games) during childhood, most commonly in the distal forearm [4]. There is recent evidence to suggest that a childhood fracture is a predictor of lower adult bone mass and increased risk of fracture in adulthood [7,8].

Recently, we have shown that children with low-energy fractures have statistically significantly lower circulating vitamin D levels compared to children without fractures [9,10], and our data indicate that higher levels of vitamin D reduce the risk of fracture [10]. Both vitamin D and vitamin K are fat-soluble vitamins. Vitamin D3 stimulates the synthesis of osteocalcin whereas vitamin K ensures full activity by facilitating carboxylation [11]. Moreover, the beneficial effects of vitamin K and vitamin D have been shown to affect serum percentage of undercaboxylated osteocalcin in relation to bone health in young children [12]. The role of vitamin D in maintaining resilient skeletal system may be supported by the biological role of vitamin K; however, there is a scarcity of research examining effects of vitamin K status on bone health and preventing bone fractures in children and adolescent populations.

Vitamin K acts as an unequivocal cofactor for formation of the active form of osteocalcin (OC), a bone-building protein [13,14,15]. It denotes a fat-soluble vitamin that occurs in nature as phylloquinone (vitamin K1), found predominantly in dark green vegetables; and menaquinones (vitamin K2), produced by bacteria and found in animal produce, e.g., organ meats, fermented cheese, yogurt, as well as the fermented soya beans [16]. Vitamin K2 is distinguished from K1 by the presence of unsaturated side chain units varying in length from 1 to 14 repeats, hence named menaquinone-4, menaquinone-7 etc. [13,17]. The status of OC has been known as a sensitive marker of vitamin K nutritional status [17,18]. Osteocalcin can be carboxylated (cOC) in the vitamin K cycle with vitamin K being an unequivocal cofactor driving this posttranslational modification. Dietary vitamin K intake is often insufficient, resulting in uncarboxylated osteocalcin (ucOC) [19]. The majority of healthy children between 6 and 18 years old have high circulating levels of uncarboxylated osteocalcin (ucOC) with the high ratio of ucOC to the carboxylated fraction of osteocalcin (cOC) [17,19]. To the best of our knowledge, we are not aware of any information in the literature about children with low-energy fractures and their vitamin K status.

The aim of current pilot study was to compare vitamin K status in healthy children with low-energy fractures with healthy children without fractures. In addition, we investigated possible correlations of vitamin K status with levels of bone turnover markers.

## 2. Materials and Methods 

### 2.1. Study Subjects 

The study group consisted of 20 children, 5 to 15 years old (14 boys and 6 girls) hospitalized in the Department of Pediatric Orthopedics of the Medical University of Bialystok, Poland (AMB), from May to August 2016 due to low-energy fractures (Table 1). The control group included 19 children (9 boys and 10 girls), 7 to 17 years old without bone fractures, hospitalized for other reasons, i.e., diagnosis of knee ligament injuries, leg length inequality and Osgood-Schlatter disease. Subjects were included in the study if they were within normal ranges for body weight and height according to reference growth charts. Exclusion criteria were medical diseases, such as metabolic, gastrointestinal, or endocrine diseases; and current medication, such corticosteroids and oral anticoagulants. Low-energy fracture was defined as a fracture sustained from a fall from the patient’s own height or a fall during team games. All fractures were radiologically confirmed.

The Bioethical Committee of the Medical University of Białystok approved the study. Written informed consent was obtained from parents of all children.

After blood sampling, all serum samples were frozen and kept at −80 °C until use. Two biochemical markers of bone turnover were determined, bone alkaline phosphatase (BALP) as marker of bone formation), and cross-linked N-terminal telopeptide of collagen (NTX) as a marker of bone resorption. BALP and NTX were measured by ELISA kits (Wuhan Fine Biological Technology Co., Ltd., Wuhan, China). Total vitamin D (25(OH)D3 plus 25(OH)D2) was determined by electrochemiluminescence using paramagnetic particles coated with streptavidin and ruthenium compound on the Cobas e 411 apparatus (Roche, Poland) [10]. To estimate bone vitamin K status, uncarboxylated (ucOC) and carboxylated (cOC) fractions of osteocalcin in serum were measured. Determination of ucOC and cOC were performed by ELISA Kit (TAKARA BIO Inc., Shiga, Japan). All assays were performed in duplicates. The UCR (ratio between ucOC and cOC) was calculated as a sensitive indicator of bone vitamin K status [16,17].

### 2.2. Statistical Analysis

Normality of distribution for all subjects was checked by Shapiro-Wilk test and histogram for all study parameters. Nonparametric Mann-Whitney test was used for comparison of parameters between fracture and non-fracture groups. Median and quartiles were used for presentation characteristic of analyzed parameters. Logistic regression analysis was used to create univariate model showing the influence of parameter UCR for risk fracture. Spearman correlation analysis was used to investigate correlations between bone markers (BALP, NTX) and indicators of vitamin K status (ucOC, cOC). A *p*-value < 0.05 was considered to be statistically significant. Statistica 12.0 software (StatSoft, Tulusa, OK, USA) was used for all analyses.

## 3. Results

### 3.1. Bone Turnover Parameters

Baseline characteristics of all subjects are shown in Table 1. The levels of BALP and NTX did not differ significantly in children with fractures compared with control children without fractures. However, it should be noted that BALP was lower in the fracture group as compared to the control group and reached borderline significance (*p* = 0.06; Table 1).

### 3.2. Vitamin K Status

**Figure 1:**
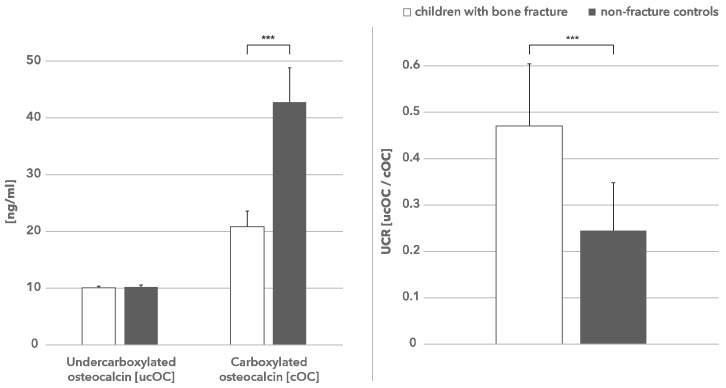
Undercarboxylated and carboxylated osteocalcin in children with bone fracture and non-fracture controls. The carboxylated osteocalcin is significantly (*** *p* < 0.0001) decreased in children with bone fractures (left hand figure) which is reflected by a higher UCR ratio (right hand figure).

The level of ucOC in both study groups was not significantly different; the median value for children with fractures was 10.11 ng/mL and for the control group 10.25 ng/mL (Table 1). However, the level of cOC was significantly decreased in children with fractures (median–20.87 ng/mL) compared with the control group (median–42.76 ng/mL) (Table 1, Figure 1).

### 3.3. UCR as Predictor of Risk Fracture

The median UCR in the fracture group was 0.471 and significantly higher compared to the control group (0.2445; *p* < 0.0001) (Table 1, Figure 1). In logistic regression analysis, the odds ratio of low-energy fractures for UCR was calculated, with an increased risk of fractures of some 78.3 times.

### 3.4. Correlation of Vitamin K Status and Bone Turnover Parameters

Bone markers BALP and NTX did not significantly correlate with ucOC, cOC, and UCR.

### 3.5. Vitamin D Status

The reference value range for total vitamin D was 30.0–74.0 ng/mL [10]. Vitamin D levels and calcium serum levels in subjects with low-energy fractures did not differ statistically significantly from subjects without fractures (data not shown) but were lower than the reference value and thus both groups were vitamin D deficient.

## 4. Discussion

Frequent fractures or low-energy fractures in children may be a sign of impaired bone health. It has been a subject of on-going discussion how fracture-prone children should be assessed by laboratory data [20]. Fractures have been reported to be associated with low bone mineral density [21,22] and certain lifestyle factors, including low calcium intake [23] or poor vitamin D status [9,10,24]. Our earlier study was designed to determine the impact of vitamin D serum levels and vitamin D receptor polymorphisms on the occurrence of low-energy fractures in children [10]. We found that vitamin D deficiency is an independent risk factor for fractures in children. In the current study, the group size was too small to draw conclusions on vitamin D difference between the fracture and the control group. Moreover, in the previous study, levels of vitamin D were low in both groups, and thus statistical significance could not be reached.

Associations between biochemical measures of vitamin K status and bone health in observational studies are relatively well recognized in adults; however, these associations are less studied and understood in the pediatric population [25]. In a study of healthy girls, 3 to 16 years old, high plasma levels of phylloquinone and low levels of ucOC were associated with lower bone turnover, but not consistently associated with high bone mineral content (BMC) [26]. In another study of healthy peri-pubertal girls, 11 to 12 years old, a better vitamin K status, as indicated by low levels of ucOC, positively correlated with bone mineral content (BMC), but not with bone turnover [12]. In another study of healthy children, 8 to 14 years old, a marked elevation of the ratio of uncarboxylated to carboxylated osteocalcin (UCR) was found. This difference persisted after adjusting for age, gender, puberty, height, and weight. In addition, a marked correlation between the bone markers for bone metabolism and levels of osteocalcin carboxylation in this group was found [27].

Levels of circulating uncarboxylated osteocalcin in healthy children and adolescents are often high in comparison to adults, suggesting that vitamin K requirement during bone development is increased and underserved in that former group [28,29,30]. The higher metabolic activity of growing bone and decline of dietary intake of vitamin K probably accounts for the vitamin K deficiency in children and adolescents, affecting integrity of the bone and making it prone to fractures. Based on a recent study, healthy children and adults above 40 years old were identified with vitamin K deficiency justifying need for vitamin K supplementation [31]. The question remains, however, whether children would benefit from higher vitamin K intake by improved bone health or stronger bones. It has been reported that six weeks daily supplementation with vitamin K2, MK7 in prepuberal children, improved UCR significantly providing evidence that vitamin K impacts bone [28].

The present study is the first to show a significantly lower fraction of carboxylated osteocalcin in children with low-energy fractures (20.87 ng/mL) as compared to the control group without fractures (42.76 ng/mL). This suggests that dietary vitamin K intake in children with low-energy fractures may be insufficient to maintain a healthy and resilient skeleton in this population of children. In logistic regression analysis, the odds ratio of low-energy fractures for UCR was calculated for fracture and non-fracture groups. An increased UCR increases the risk of fracture by some 78.3 times. The UCR is highly sensitive for assessing bone vitamin K status and in clinical practice it may serve to monitor treatment of patients with vitamin K deficit and detect a preventable adverse lifestyle factors [28].

Our study illustrates the potential consequences of vitamin K deficiency in growing bone. A limitation of this study is the small sample of studied population. We did not perform a power calculation but included all children that were hospitalized in the period May–August 2016 in this pilot study. The results of this study did not show a correlation of vitamin K-deficiency with markers of bone metabolism (BALP and NTX); however, it should be noted that BALP was different between fracture and non-fracture groups which was borderline significant (*p* = 0.06). A more definite confirmation that low levels of osteocalcin carboxylation may affect bone quality in a pediatric population need to be established in a large sample prospective study. Another limitation is the lack of dietary vitamin K intake. It is known that intake of vitamin K varies and influences carboxylation of vitamin K-dependent proteins. In future studies, food frequency questionnaires (FFQ) should be taken into account when measuring effects of osteocalcin carboxylation on low-energy bone fractures.

Based on our pilot study, we hypothesize that besides vitamin D, vitamin K plays an important role in bone health in children. This indicates potential benefits of using supplementation of vitamin D and vitamin K in children to prevent low-energy bone fractures.

## Figures and Tables

**Table 1:** Demographic and anthropometric data, bone markers, and vitamin K parameters of children with fracture and non-fracture.

Parameter	Fracture (*n* =20)		Non-Fracture (*n* = 19)		*p*
	Median (Q2)	Q1–Q3	Median (Q2)	Q1–Q3	
**Age (years)**	12.0	8.0–13.0	13.5	11.0–15.5	0.08
**Boys**					
*n*	14		9		
**Girls**					
*n*	6		10		
**Weight (kg)**	45.7	26.3–55.5	60.5	41.0–67.0	0.04
**Bone markers**					
BALP, ng/mL	37.19	30.75–53.30	50.75	36.39–114.5	0.06
NTX, ng/mL	0.483	0.438–0.546	0.523	0.469–0.732	0.13
**Undercarboxylated osteocalcin (ucOC), ng/mL**					
Median	10.104	9.356–10.350	10.252	9.677–10.552	0.16
**Carboxylated osteocalcin (cOC), ng/mL**					
Median	20.866	16.390–23.612	42.762	29.980–48.808	<0.0001
**UCR (ucOC/cOC)**	0.471	0.404–0.605	0.245	0.202–0.348	<0.0001
**Vitamin D** **(reference value range 30–74 ng/mL)**	18.00	10.00–30.00	25.00	15.50–32.50	0.22
**Concentration Ca mmol/L** **(reference value range 1.10–150 mmol/L)**	1.25	1.18–1.36	1.23	1.23–1.32	0.81

Assessment of vitamin K status and bone turnover markers. BALP; Bone alkaline phosphatase, NTX; N-terminal telopeptide, UCR; Uncarboxylated osteocalcin ratio, Q1–Q3; quartile 1 to quartile 3. Median is referred to Q2.
